# Ventral Midbrain NTS1 Receptors Mediate Conditioned Reward Induced by the Neurotensin Analog, D-Tyr[11]neurotensin

**DOI:** 10.3389/fnins.2015.00470

**Published:** 2015-12-22

**Authors:** Khalil Rouibi, Poulomee Bose, Pierre-Paul Rompré, Richard A. Warren

**Affiliations:** ^1^Department of Neurosciences, Université de MontréalMontréal, QC, Canada; ^2^FRQ-S Research Group in Behavioral Neurobiology, Department of Psychology, Concordia UniversityMontréal, QC, Canada; ^3^Department of Psychiatry, Faculty of Medicine, Université de MontréalMontréal, QC, Canada

**Keywords:** conditioned reward, glutamate, neurotensin, ventral midbrain

## Abstract

The present study was aimed at characterizing the mechanisms by which neurotensin (NT) is acting within the ventral midbrain to induce a psychostimulant-like effect. In a first experiment, we determine which subtype(s) of NT receptors is/are involved in the reward-inducing effect of ventral midbrain microinjection of NT using the conditioned place-preference (CPP) paradigm. In a second study, we used *in vitro* patch clamp recording technique to characterize the NT receptor subtype(s) involved in the modulation of glutamatergic neurotransmission (excitatory post-synaptic current, EPSC) in ventral tegmental neurons that expressed (${\text{I}}_{\text{h}}^{+}$), or do not express (${\text{I}}_{\text{h}}^{-}$), a hyperpolarization-activated cationic current. Behavioral studies were performed with adult male Long-Evans rats while electrophysiological recordings were obtained from brain slices of rat pups aged between 14 and 21 days. Results show that bilateral ventral midbrain microinjections of 1.5 and 3 nmol of D-Tyr[^11^]NT induced a CPP that was respectively attenuated or blocked by co-injection with 1.2 nmol of the NTS1/NTS2 antagonist, SR142948, and the preferred NTS1 antagonist, SR48692. In electrophysiological experiments, D-Tyr[^11^]NT (0.01-0.5 μM) attenuated glutamatergic EPSC in ${\text{I}}_{\text{h}}^{+}$ but enhanced it in ${\text{I}}_{\text{h}}^{-}$ neurons. The attenuation effect (${\text{I}}_{\text{h}}^{+}$ neurons) was blocked by SR142948 (0.1 μM) while the enhancement effect (${\text{I}}_{\text{h}}^{-}$ neurons) was blocked by both antagonists (0.1 μM). These findings suggest that (i) NT is acting on ventral midbrain NTS1 receptors to induce a rewarding effect and (ii) that this psychostimulant-like effect could be due to a direct action of NT on dopamine neurons and/or an enhancement of glutamatergic inputs to non-dopamine (${\text{I}}_{\text{h}}^{-}$) neurons.

## Introduction

Neurotensin (NT), a tridecapeptide (pGlu-Leu-Tyr-Glu-Asn-Lys-Pro-Arg-Arg-Pro-Tyr-Ile-Leu-OH) isolated from the hypothalamus more than four decades ago (Carraway and Leeman, 1973), acts as a potent modulator of limbic neurotransmission. Cell bodies and terminals that express NT-like immunoreactivity are found in several limbic brain regions including the amygdala, the nucleus accumbens, the prefrontal cortex, the septum, and the ventral midbrain (Jennes et al., 1982; Hökfelt et al., 1984; Woulfe and Beaudet, 1989; Delle Donne et al., 1996). When released from nerve terminals, NT can activate three receptor sub-types, NTS1, NTS2, and NTS3 (see Vincent et al., 1999). The NTS1 and NTS2 are metabotropic receptors that are coupled to G-proteins linked to different signaling pathways such as cyclic guanosine-monophosphate, phospholipase C and mitogen-activated protein kinase. The NTS3 is a non G- protein coupled receptor that possesses a single transmembrane domain; this receptor appears to be non-selective for NT as its binds several other endogenous ligands (see Mazella and Vincent, 2006). The great majority of the central effects of NT have been attributed to its action on either NTS1 or NTS2 receptors. When administered into the lateral ventricle, for instance, NT produces a dose-dependent hypothermia and analgesia that are prevented by the NTS1/NTS2 antagonist, SR142948, but not the preferred NTS1 antagonist, SR48692, suggesting that they are mediated by the NTS2 receptor (Gully et al., 1997). Central NT injections also attenuate spontaneous and methamphetamine-induced locomotor activity, effects that are prevented by SR48692 suggesting that they are mediated by the NTS1 receptor (Wagstaff et al., 1994; Gully et al., 1995). The behavioral effects of NT are not only dependent upon the receptor sub-type that is activated but also upon the site of action of the peptide within the limbic system. In the ventral midbrain, for instance, NT stimulates dopamine impulse flow and dopamine-dependent behaviors (Kalivas et al., 1981; Holmes and Wise, 1985; Rompré et al., 1992) while in the ventral striatum and the prefrontal cortex, it reduces the post-synaptic effect of dopamine and attenuates dopamine-dependent behaviors (Ervin et al., 1981; Kalivas et al., 1984; Béauregard et al., 1992; Stowe et al., 2005). These findings led to the hypothesis that NT may act as either an endogenous antipsychotic- or psychostimulant-like neuromodulator (Bérod and Rostène, 2002; Kinkead and Nemeroff, 2002). The mechanisms by which NT produces psychostimulant-like effects remain imprecise. The main hypothesis is that NT enhances dopamine release and dopamine-dependent behaviors by stimulating dopamine impulse flow through activation of NTS1 receptors expressed on dopamine cell bodies and dendrites (see Bérod and Rostène, 2002). Consistently, NT induces an increase in dopamine inward current and firing rate that is prevented by SR48692 (St-Gelais et al., 2006). Activation of NTS1 receptors expressed on dopamine neurons also inactivates the dopamine auto-receptor which contributes to enhance dopamine impulse flow (Thibault et al., 2011). We, and others, also reported that NT and its C-terminal fragment, NT-(8-13), enhance excitatory post-synaptic currents (EPSCs) in presumed dopamine neurons, an effect that is blocked by SR48692 (Kempadoo et al., 2013; Bose et al., 2015). Unexpectedly, the increase in ventral striatal dopamine release induced by ventral midbrain application of NT is blocked by SR142948 but not by SR48692 (Steinberg et al., 1994; Leonetti et al., 2002) suggesting that NT is rather stimulating dopamine impulse flow through activation of NTS2 receptors. This latter finding, however, was not supported by another study showing that application of ventral midbrain NT enhances ventral striatal dopamine release in NTS2 but not NTS1 knock-out mice (Leonetti et al., 2004). Neurotensin structure-activity studies have also generated conflicting results regarding the role of NTS1 receptors in the psychostimulant-like effect of NT. For example, the enhancement effect of NT on locomotor activity and on brain stimulation reward is mimicked by NT-(8-13) and neuromedin N (Kalivas and Taylor, 1985; Kalivas et al., 1986; Rompré and Gratton, 1992, 1993), two peptides that bind and activate the NTS1 receptor (Kitabgi et al., 1980; Tanaka et al., 1990). But the induction of a conditioned place-preference (CPP) by repeated ventral midbrain NT microinjections is not mimicked by an equimolar concentration of NT-(8-13); in fact it is mimicked by a NT fragment, NT-(1-11), that fails to interact with the NTS1 receptor (Kitabgi et al., 1980; Glimcher et al., 1984); these results suggest that the conditioned rewarding effect of NT may be mediated by a NT receptor other than NTS1 receptor. In order to clarify this issue, we attempted to determine which ventral midbrain NT receptor is involved in the induction of a CPP using the NT analog, [D-Tyr^11^]NT and the NT receptor antagonists, SR142948 and SR48692. [D-Tyr^11^]NT mimicks several behavioral and neurochemical effects of NT. When administered into the cerebral ventricle, for instance, NT and [D-Tyr^11^]NT enhances brain stimulation reward (Rompré, 1995; Bauco and Rompré, 2001) and sensitizes to the locomotor activating effect of amphetamine (Rompré, 1997). Ventral midbrain infusion of NT and [D-Tyr^11^]NT stimulates locomotor activity (Bauco and Rompré, 2003) and enhances mesoaccumbens DA release (Steinberg et al., 1995; Sotty et al., 2000). But, when injected unilaterally, [D-Tyr^11^]NT is more effective than NT at inducing circling behavior (Steinberg et al., 1995) and less effective at enhancing mesoprefontal DA release (Sotty et al., 2000). These findings are consistent with previous results showing than [D-Tyr^11^]NT is a NT agonist that may preferentially activates one sub-type of NT receptors (Kitabgi et al., 1980; Labbé-Jullié et al., 1994); it thus constitutes an useful pharmacological tool to sort out the role of each of these receptors in behavior. Because NT and NT-(8-13) also enhance ventral midbrain glutamatergic neurotransmission, we characterized the effect of [D-Tyr^11^]NT on glutamatergic EPSCs in putative ventral midbrain dopamine and non-dopamine neurons; the two populations were distinguished by the presence, or the absence, of a hyperpolarization-activated cationic current (Margolis et al., 2006) using the patch clamp recording technique. Results of the behavioral experiments show that [D-Tyr^11^]NT induced a dose-dependent CPP that was blocked by SR48692 and attenuated by SR142948, suggesting that it is mediated by NTS1 receptors. Electrophysiological results show that [D-Tyr^11^]NT dose-dependently attenuates glutamatergic EPSCs in putative dopamine neurons while it enhances the EPSCs amplitude in non-dopamine neurons; these effects are likely mediated by a respective activation of NTS2 and NTS1 receptors.

## Materials and methods

### Behavioral experiments

#### Animals

Male Long-Evans rats (Charles River, St-Constant, Qc, Canada) weighing 280–320 g at the time of surgery were used. They were housed 1 (after surgery) or 2 per cage in a temperature (22 ± 1°C) and humidity (40–50%) controlled room with a 12 h light/dark cycle (lights on 06:00); standard rat chow and water were available *ad libitum*. All testing was performed during the light phase of the light–dark cycle. All animal experimental procedures were approved by the Institutional Animal Ethics Committee (Comité de déontologie de l'expérimentation sur les animaux de l'Université de Montréal), in accordance with the Guide for the Care and Use of Laboratory Animals published by the US National Institutes of Health (n°: 85–23, revised 1996). All efforts were made to minimize the suffering and number of animals used.

#### Surgery

Following 1 week habituation period to the colony room, each rat was anesthetized with isoflurane (2.5–3.5%, 0.75 L/min O_2_); solutions of 0.1 ml of Anafen (5 mg/kg, s.c.) and 0.05 ml (i.m.) of duplocillin LA containing 15,000 I.U. of penicillin were administered to prevent inflammation and infection. The animals was then mounted on a stereotaxic apparatus, the surface of the skull was exposed and a guide cannula (Model C315G, Plastic One, VA, USA,) was implanted in each hemisphere, above the ventral tegmental area (VTA), using the following stereotaxic coordinates: 5.5 mm posterior to bregma, 1.7 mm lateral and 6.3 mm below the surface of the cranium (Paxinos and Watson, 1986); cannulae were inserted into the brain with a mediolateral angle of 8° and were closed with an obturator of the same length. Four stainless-steel screws were threaded into the bone and the cannulae were anchored to the skull with dental acrylic. Behavioral tests started 1 week after the surgery.

#### Conditioned place preference (CPP) paradigm

The CPP apparatus (Med Associates, St. Albans, VT, USA) consisted of a rectangular Plexiglas box divided into two large compartments (26 × 21 × 21 cm) separated by a smaller central compartment (21 × 12 × 21 cm). Two sliding doors separated the central gray compartment from the two others which have distinct wall colors (white or black) and floors (grid or bar). Locomotor activity and times spent in each chamber were measured by computer-interfaced infrared photobeams (Med Associates, St. Albans, VT, USA). The CPP experiment lasted 10 days and consisted of a habituation phase, a conditioning phase and a test phase. On the first day of the habituation phase, rats were allowed to explore the entire CPP apparatus for 20-min to reduce neophobia. On day 2, all animals received a first intra-VTA injection of 0.5 μl/side of saline and were allowed to explore freely the entire CPP apparatus for 20-min. On the third day, animals were allowed to explore the entire CPP apparatus for 20-min and time spent in each of the two large compartments was measured; rats exhibiting higher or lower time interval than 20–80% of time in a compartment were excluded (unbiased procedure). Conditioning began the next day. Conditioning trials lasted 30-min and were conducted daily for 6 days. Control and drug treatment groups were conditioned in either the black or the white compartment of the apparatus. On the first day of the conditioning phase, the drug-conditioned animals were injected in the VTA with vehicle and were immediately placed into one compartment of the apparatus for 30 min. The next day, animals were injected with [D-Tyr^11^]NT (1.5 or 3 nmol/0.5 μl/side), SR142948 (1.2 nmol/0.5 μl/side), SR48692 (1.2 nmol/0.5 μl/side), [D-Tyr^11^]NT (3 nmol/0.5 μl/side) + SR142948 (1.2 nmol/0.5 μl/side) or [D-Tyr^11^]NT (3 nmol/0.5 μl/side) + SR48692 (1.2 nmol/0.5 μl/side) and were immediately placed into the other compartment of the apparatus for 30 min. This procedure was repeated three times so that rats received three vehicle (Day 4, 6, and 8) and three drug (Day 5, 7, and 9) injections. Animals in the control group were injected with the vehicle on each day and were similarly conditioned for 6 days. Twenty four hours after the last day of the conditioning phase, on day 10, animals were allowed to explore the apparatus for 20 min and the time spent in each compartment was measured. Animals were tested between 11:00 and 17:00 under an ambient light intensity of 5 lux and were habituated to the experimental room for 1-h prior to the behavioral testing.

#### Microinjection procedure

Bilateral microinjections were made by inserting into each guide cannula an injection cannula (model C315I) that extended 2 mm beyond the tip of the guide. Each cannula was connected with polyethylene tubing to a 2-μl microsyringe and a volume of 0.5 μl of solution was injected into each hemisphere simultaneously with a micro-infusion pump over a period of 60 s; cannulae were left in place for an additional 60 s to allow diffusion into the surrounding brain tissue.

#### Histology

At the end of the experiment, animals were deeply anesthetized with urethane (2 g/kg, i.p.) and transcardially perfused with 0.9% saline followed by 10% formalin. Brains were removed, stored in 10% formalin and subsequently sliced in serial 40-μm sections that were stained with formal-thionin solution. Locations of the injection sites were determined under light microscopic examination. Only animals that had both injection sites within the VTA, including the rostral and caudal linear nuclei, the paranigral, parabrachial, and the interfascicular nuclei between 5.0 and 6.0 mm behind bregma (Paxinos and Watson, 1986) were included in the analyses.

#### Drugs

[D-Tyr^11^]neurotensin-(1-13) was purchased from Bachem (Sunnydale, CA, USA) and dissolved in sterile 0.9% saline at a concentration of 3 or 6 nmol/μl. The neurotensin antagonist, SR-142948 and SR-48692 were purchased from Tocris Bioscience (Burlington, ON, Canada) and were dissolved at a concentration of 2.4 nmol/μl in a sterile 0.9% sodium chloride solution that contained 20% dimethylsufoxyde (DMSO). All solutions were stored at −20°C in 50 μl aliquots in silicone-coated tubes; they were thawed just before testing and were used only once.

#### Statistical analysis

Preference score was determined by subtracting the time spent in the drug-paired compartment measured before the conditioning phase (Pre) to the time spent in the same compartment measured on the conditioning test day (Post). Preference score and locomotor activity (horizontal and stereotypic-like movements) measured during the conditioning test day were analyzed with a One-way analysis of variance (ANOVA). The Duncan's multiple range *post-hoc* tests was used for individual group comparisons. The accepted value for significance was set at 0.05 (Statistica V5.0, StatSoft).

### Electrophysiological experiments

#### Animals and slice preparation

Fourteen to 21-day-old (P14-P21) Long Evans pups of either sex obtained from Charles River (St-Constant, QC) were used. Pups were anesthetized by methoxyflurane vapor inhalation in a closed chamber, decapitated and their brain quickly removed and transferred to chilled, oxygenated artificial cerebrospinal fluid (ACSF) in which NaCl had been replaced by equivalent osmolarity of sucrose and containing (in mM) sucrose 252 (NaCl 126 in standard ACSF); KCl, 3; NaH_2_PO_4_, 1.25; MgSO_4_ 7 H_2_O, 1.3; CaCl_2_, 2.5; NaHCO_3_, 26; and glucose, 10, and saturated with a gas mixture of 95% O_2_ and 5% CO_2_. Two hundred and fifty micrometer thick horizontal slices preserving the VTA afferents (Margolis et al., 2006) were cut using a vibrating microtome (DSK Microslicer). Slices were transferred to a submerged recording chamber maintained between 32 and 34°C and superfused with standard ACSF at a rate of 2 ml/min; slices were incubated for at least 1 h before recording began.

#### Electrophysiological recordings

Whole-cell configuration was achieved using the “blind” patch-clamp technique (Blanton et al., 1989). Pipettes were pulled from thin wall borosilicate capillary glass on a P-87 micropipette puller (Sutter Instrument, Novato, CA, USA). Recording pipettes had a resistance of 3–5 MΩ when filled with a solution containing (in mM) potassium gluconate, 140; MgCl_2_, 2; CaCl_2_, 0.1; EGTA, 1.1; HEPES, 10; K_2_-adenosine trisphosphate (ATP), 2; guanosine trisphosphate (GTP), 0.5 and biocytin (5%). The pH was adjusted to 7.3 with KOH solution, and final osmolarity was 280 ± 5 mosmol/kg. Biocytin (5%) was added in the recording pipette and all recorded cells were processed after recording to confirm their location in the medial VTA.

Whole-cell recordings were made with an Axoclamp 2B amplifier (Molecular Devices, Sunnyvale, CA, USA) in continuous single-electrode voltage-clamp mode. The output of the amplifier was fed into a LPF 200A DC amplifier/filter (Warner Instruments Corp., Hamden, CT, USA) and digitized at 5–10 kHz with a real-time acquisition system (CED 1401 Power). Data acquisition was achieved using the Signal 4.0 software (Cambridge Electronic Design, Cambridge, England). Recording pipette's capacitance was optimally adjusted before whole-cell configuration was achieved. The resting membrane potential was measured just after rupturing the cell membrane and the offset potential, measured upon withdrawal of the electrode from the cell, was accounted for assuming that it drifted in a linear fashion with time from the start of the recording session. We did not correct for liquid junction potential which for a pipette containing 140 mM potassium gluconate amounts for an additional potential shift of around −10 mV (Spigelman et al., 1992).

#### Synaptic activation and drug application

The presence of I_h_ current was determined by voltage clamping cells at −60 mV and stepping to −40, −50, −70,−80, −90, −100, −110, and −120 mV. Input resistance was monitored with hyperpolarizing pulses in current clamp mode. A monopolar tungsten stimulating microelectrode was placed rostral to the recording site in the medial VTA, on the slice superficial layer, 0.5–1.0 mm from the recording electrode. Excitatory postsynaptic currents were evoked by 0.1 ms, 3–6 V cathodal pulses delivered at 15 sec intervals. In order to isolate glutamate receptor-mediated EPSCs, all experiments were performed in the presence of (−) bicuculline methiodide (BMI, 10 μM) in bath solution to block GABA_A_ receptor-mediated synaptic currents. BMI was applied 30 min before obtaining whole-cell configuration to ensure a complete diffusion in the slice tissue. In all experiments the EPSCs were recorded from an online voltage-clamped potential of −70 mV. The effects of D-Tyr [11] NT on glutamatergic EPSCs were assessed at a holding membrane potential of −70 mV. Three concentrations of the peptide (0.01, 0.1, and 0.5 μM) were tested, one concentration per cell. Upon agonist application, the change in amplitude of the glutamatergic EPSC was measured. Five minutes of baseline EPSC activity was recorded before superfusion with the peptide. The EPSC amplitudes were recorded during 7 min after the onset of the peptide application and averaged over the last 5 min. A washout period of 15 min was allowed before the amplitude of the recovered EPSC was measured. In some experiments, the control EPSC amplitude was measured for 4 min before a NT antagonist was added to the superfusion medium. SR142948 nor SR48692 produced any change in EPSC amplitude (*n* = 12; data not shown); therefore in further experiments where NTS receptor antagonists were used, SR142948 or SR48692 was added to the superfusing medium for 7 min and a control response was measured in the presence of the antagonist.

#### Drugs and peptides

The following pharmacological agents were applied through the superfusing ACSF: (−) bicuculline methiodide obtained from Sigma Aldrich (Oakville, Ontario, Canada); D-Tyr [11] NT from Bachem (Sunnyvale, CA, USA); SR-48692 and SR1429482 obtained from Tocris Biosciences (Burlington, ON, Canada). All drugs were made up as 10 mM stock solutions in distilled water and diluted with ACSF solution to final concentration just before addition to the perfusion medium with the exception of SR48692 which was dissolved in DMSO (final concentration 0.1%) and distilled water.

#### Data analysis

Data analysis was performed using Signal software (Cambridge Electronic Design, Cambridge, England). The magnitude of EPSC recorded after application of the peptide was expressed as percent of baseline and group means were calculated for drug condition. A One-way ANOVA was performed and Duncan *post-hoc* test used to determine significant differences between concentration or drug and peptide condition when justified; level of significance was set at 0.05 (Statistica V5.0, StatSoft).

## Results

### Behavioral experiment

From the 86 rats that completed the experiment, 10 were excluded from the analysis because the injection sites were dorsal or anterior to the VTA, or because the sites (left and right hemisphere) overlapped on the midline; an additional rat was excluded because the injection sites could not be located.

### Ventral midbrain microinjection of [D-Tyr^11^]NT induced a conditioned place preference

**Figure 1:**
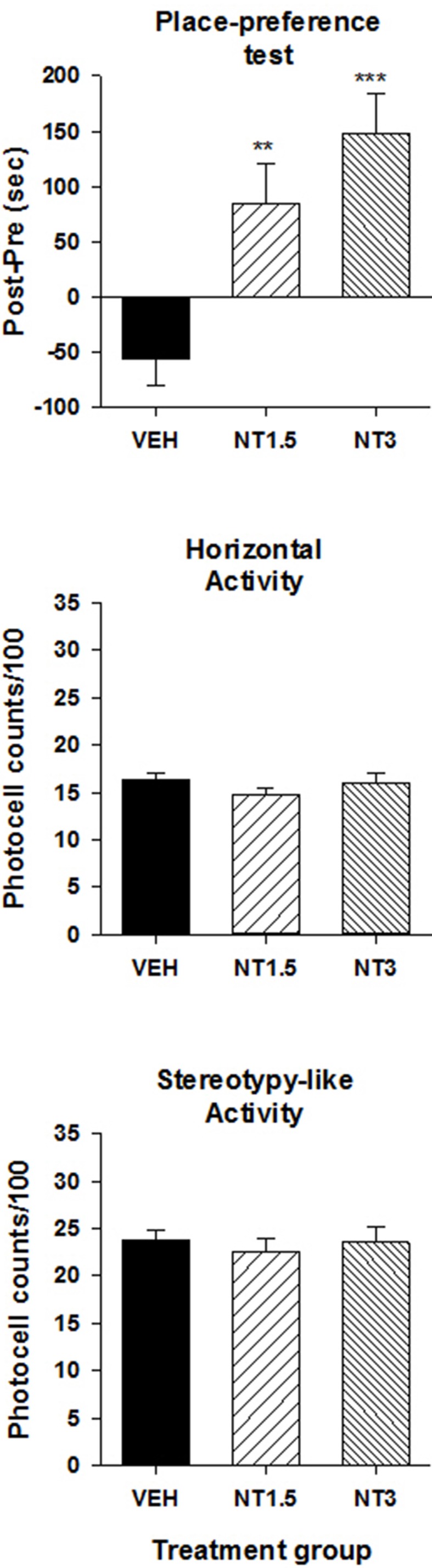
**Induction of a CPP by [D-Tyr^**11**^]NT**. **Top panel** illustrates the preference score measured on the test day for the animals that were injected with 1.5 nmol (NT1.5, *n* = 6), 3 nmol (NT3, *n* = 13) of [D-Tyr^11^]NT or its vehicle (VEH, *n* = 15). Preference score corresponds to the amount of time (in sec) spent in the paired compartment on the test day minus the time spent at baseline in the same compartment. Measures of locomotor activity recorded during the preference test for the animals in each treatment group are presented in the **middle panel** (horizontal) and **bottom panel** (stereotypy-like). Asterisks indicate a statistical significant difference with VEH (^**^p < 0.01; ^***^p < 0.001).

**Figure 2:**
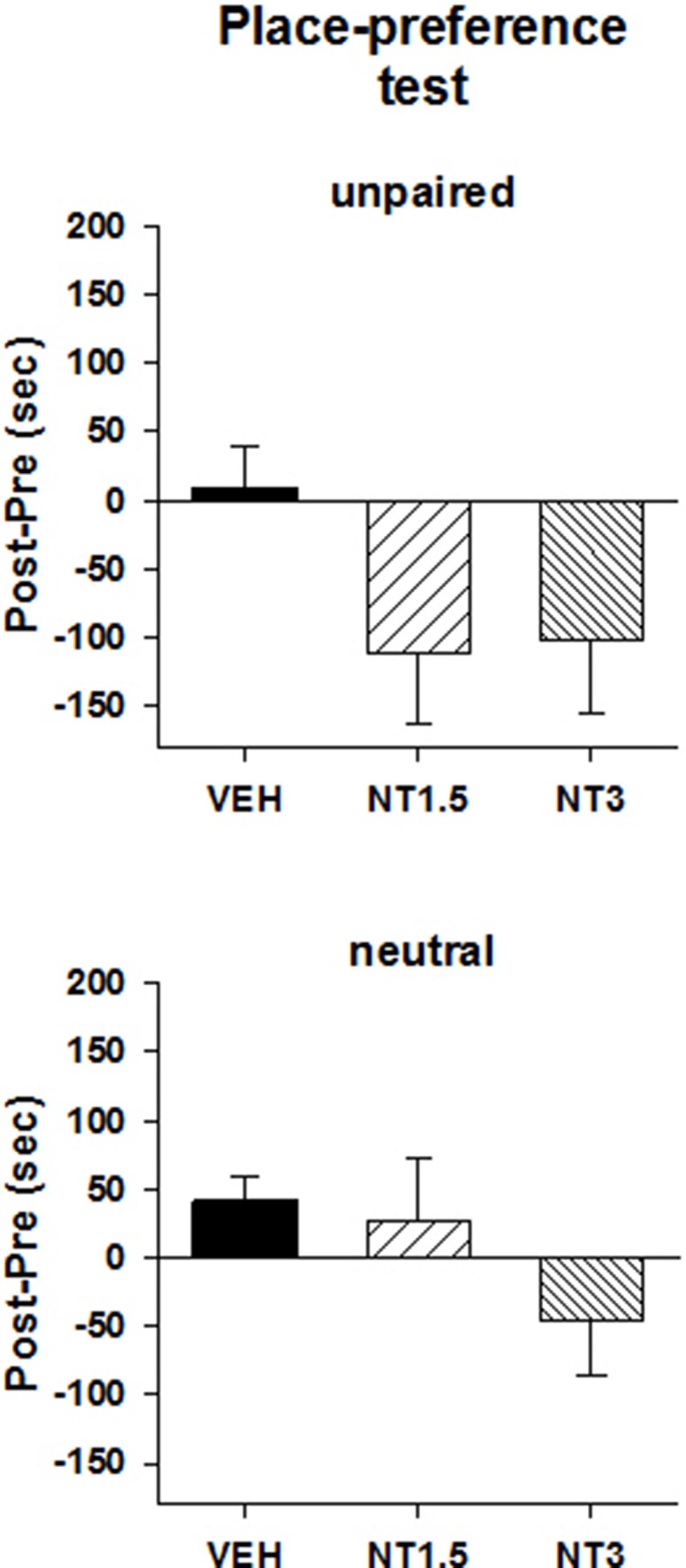
**Mean preference score measured on the test day in the unpaired (top panel) and neutral (bottom panel) compartment for the animals that were injected with 1.5 nmol (NT1.5, ***n*** = 6), 3 nmol (NT3, ***n*** = 13) of [D-Tyr^**11**^]NT or its vehicle (VEH, ***n*** = 15)**. Preference score corresponds to the amount of time (in sec) spent in the unpaired or neutral compartment on the test day minus the time spent at baseline in the same compartment. See text for details.

Figure 1 illustrates the preference score (top panel) and locomotor activity (middle and bottom panels) measured during the conditioning test in different groups of rats that were conditioned with the vehicle and one of two doses of [D-Tyr^11^]NT. As can be seen, animals that were conditioned with VTA [D-Tyr^11^]NT microinjections spent more time in the peptide associated compartment than the animal conditioned with VTA microinjections of the vehicle. The ANOVA yielded a significant effect of treatment [*F*_(2, 31)_ = 13.1, *p* < 0.001] and *post-hoc* test showed that preference score of each [D-Tyr^11^]NT group was significantly different than vehicle; although the preference score for the group treated with the highest dose was superior to that of the lower dose there was no significant difference between the two doses (*p* > 0.05). In order to determine whether the preference for the [D-Tyr^11^]NT-paired compartment was in part related to a conditioned aversion to the unpaired compartment, we compared the preference score for this compartment and the neutral compartment among the three groups. Animals that were injected with [D-Tyr^11^]NT spent less time in the unpaired compartment on the conditioned test day than those injected with the vehicle (Figure 2, top panel) but the ANOVA yielded no significant effect of treatment [*F*_(2, 31)_ = 2.4, *p* > 0.05]. Moreover, the animals conditioned with the high dose of [D-Tyr^11^]NT spent slightly less time in the neutral compartment (Figure 2, bottom panel) compared to the other groups but the difference was not significant [*F*_(2, 31)_ = 2.33, *p* > 0.05]. Altogether, these results suggest that the reduction in the time spent in the compartment non-associated with VTA [D-Tyr^11^]NT in the conditioned groups was due to an increase in the amount of time spent in the conditioned but not the neutral compartment, hence confirming the occurrence of a conditioned preference effect.

The overall locomotor activity (in the entire apparatus) measured during the conditioned test did not differ between groups suggesting that repeated exposure to VTA [D-Tyr^11^]NT did not induce conditioned locomotor activity (Figure 1, middle and bottom panels). The ANOVA performed on each measure of activity, horizontal and stereotypy-like, yielded no significant effect of treatment [horizontal activity, *F*_(2, 31)_ = 0.63 *p* > 0.05; stereotypy-like activity, *F*_(2, 31)_ = 0.14, *p* > 0.05].

### [D-Tyr^11^]NT-induced a conditioned place preference: role of NTS1 receptors

**Figure 3:**
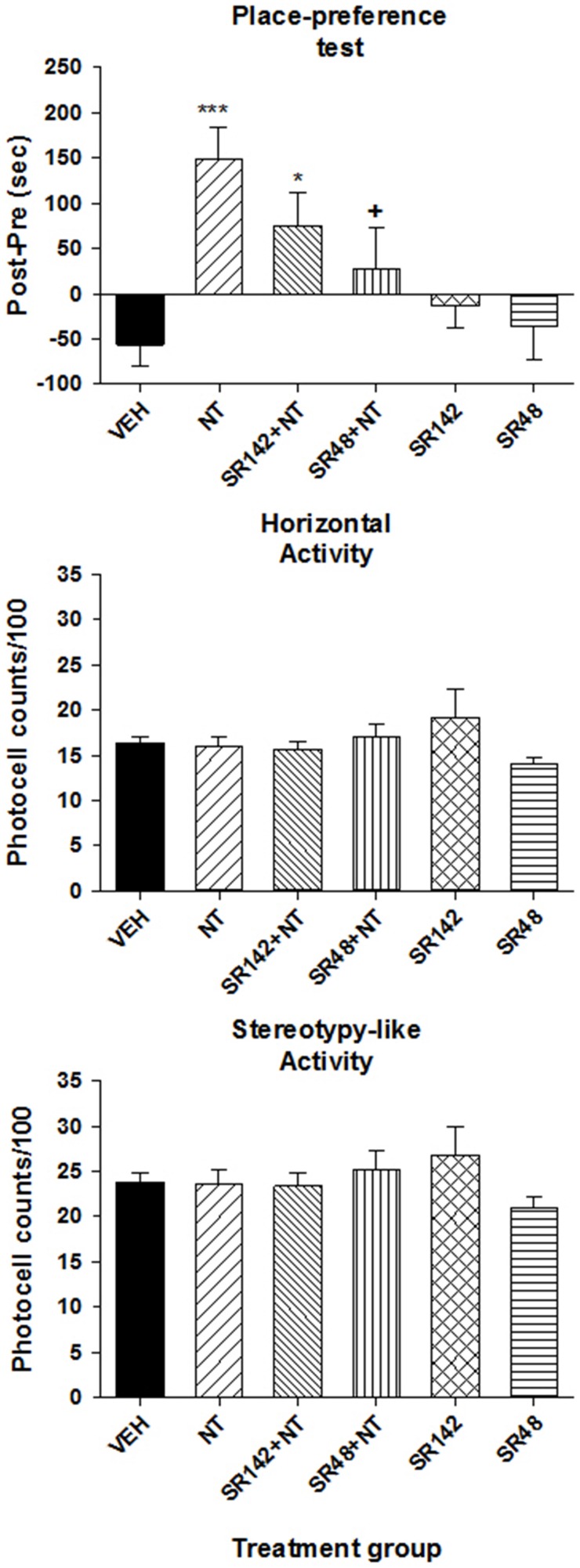
**Effects of SR142948 and SR48692 on [D-Tyr^**11**^]NT-induced CPP**. **Top panel** illustrates the preference score measured on the test day for the animals that were injected with 3 nmol of [D-Tyr^11^]NT (NT, *n* = 13), 1.2 nmol of SR142948 (SR142, *n* = 10), 1.2 nmol of SR48692 (SR48, *n* = 7), SR142948 + [D-Tyr^11^]NT (SR142+NT, *n* = 12), SR48292 + [D-Tyr^11^]NT (SR48+NT, *n* = 10) or the vehicle (VEH, *n* = 15). Preference score corresponds to the amount of time (in sec) spent in the paired compartment on the test day minus the time spent at baseline in the same compartment. Measures of locomotor activity recorded during the preference test for the animals in each treatment group are presented in the **middle panel** (horizontal) and **bottom panel** (stereotypy-like). The asterisks and the cross indicate a statistical significant difference with VEH (^*^p < 0.01; ^***^p < 0.001) and NT (^+^*p* < 0.05) respectively.

To determine which NT receptor is involved in the induction of a CPP by VTA [D-Tyr^11^]NT, we compared the preference score obtained from animals that were conditioned with vehicle and [D-Tyr^11^]NT alone to that of animals conditioned with either SR142948 with [D-Tyr^11^]NT or SR48692 with [D-Tyr^11^]NT, or each NT antagonist alone. Results presented in Figure 3 (top panel) shows that SR48692 blocked the induction of a CPP. The ANOVA yielded a significant effect of treatments [*F*_(5, 61)_ = 5.74, *p* < 0.001] and *post-hoc* test showed that the preference score of the group injected with preferred NTS1 antagonist, SR48692, with [D-Tyr^11^]NT is not significantly different than that of the vehicle injected animals but is significantly different than that of the [D-Tyr^11^]NT alone injected animals. The NTS1/NTS2 antagonist, SR142948, attenuated the induction of a CPP. When administered alone during the conditioning phase, the antagonists induced no conditioned effect. Altogether, these results show that the conditioned preference is due to activation of VTA NTS1 receptors. Locomotor activity measured in the entire apparatus during the conditioned test did not differ between groups [Figure 3, middle and bottom panels; horizontal activity, *F*_(5, 61)_ = 1.14, *p* > 0.05; stereotypy-like activity, *F*_(5, 61)_ = 0.93. *p* > 0.05].

## Electrophysiological results

Whole-cell voltage-clamp recording was carried out on 96 physiologically identified VTA neurons. Neurons were designated as ${\text{I}}_{\text{h}}^{+}$ (*n* = 54) or ${\text{I}}_{\text{h}}^{-}$ (*n* = 42) based on the presence or absence of the hyperpolarization activated cationic current (I_h_). The amplitude of I_h_ in ${\text{I}}_{\text{h}}^{+}$ positive neurons ranged from 67 pA to 419 pA with an average of 192.3 pA (*n* = 54; data not shown).

### Effects of D-Tyr [11] NT on glutamatergic EPSCs in ${\text{I}}_{\text{h}}^{+}$ and ${\text{I}}_{\text{h}}^{-}$ neurons

The effects of [D-Tyr^11^]NT on glutamatergic EPSCs in VTA neurons were measured at a holding membrane potential of −70 mV upon application at three different concentrations (0.01, 0.1, and 0.5 μM). Desensitization of the response to [D-Tyr^11^]NT application allowed only one concentration of the peptide to be tested per cell.

**Figure 4:**
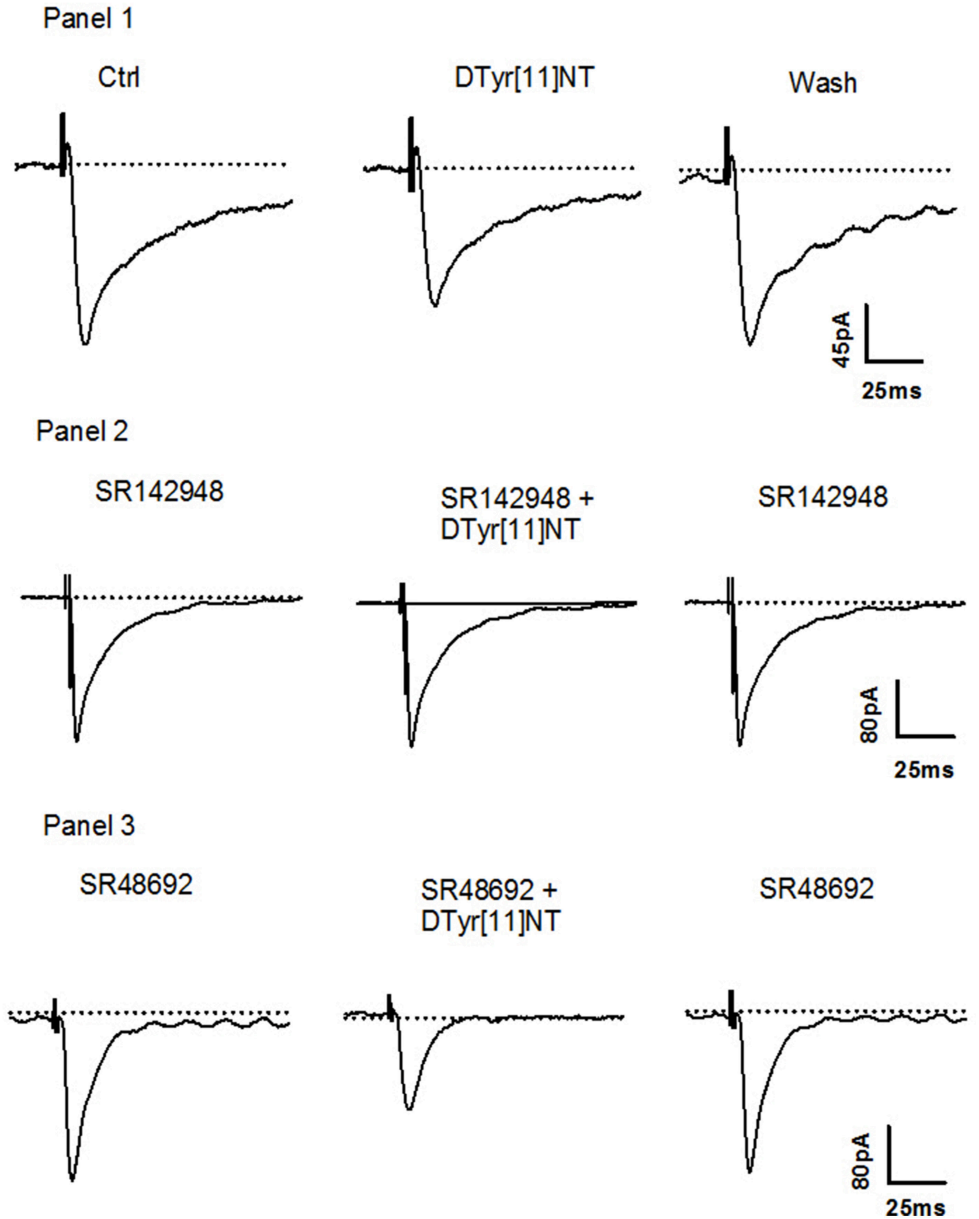
**Effect of [D-Tyr^**11**^]NT and antagonists on ${\text{I}}_{\text{h}}^{+}$ neurons**. **Panel 1:** Current traces of glutamatergic EPSC recorded during superfusion of [D-Tyr^11^]NT; control (1), with [D-Tyr^11^]NT (0.01 μM) (2) and following the washout of [D-Tyr^11^]NT (3) at a holding membrane potential of −70 mV in ${\text{I}}_{\text{h}}^{+}$ neurons (*n* = 6). **Panel 2:** Current traces of glutamatergic EPSC recorded during superfusion with SR142948 (0.1 μM) (1), with SR142948 and [D-Tyr^11^]NT (0.01 μM) (2) and with SR142948 following the washout of (3) at a holding membrane potential of −70 mV in ${\text{I}}_{\text{h}}^{+}$ neurons (*n* = 5). **Panel 3:** Current traces of glutamatergic EPSC recorded during superfusion with SR48692 (0.1 μM) (1), with SR48692 and [D-Tyr^11^]NT (0.01 μM) (2) and with SR48692 following the washout of [D-Tyr^11^]NT (3) at a holding membrane potential of −70 mV in ${\text{I}}_{\text{h}}^{+}$ neurons (*n* = 4).

**Figure 5:**
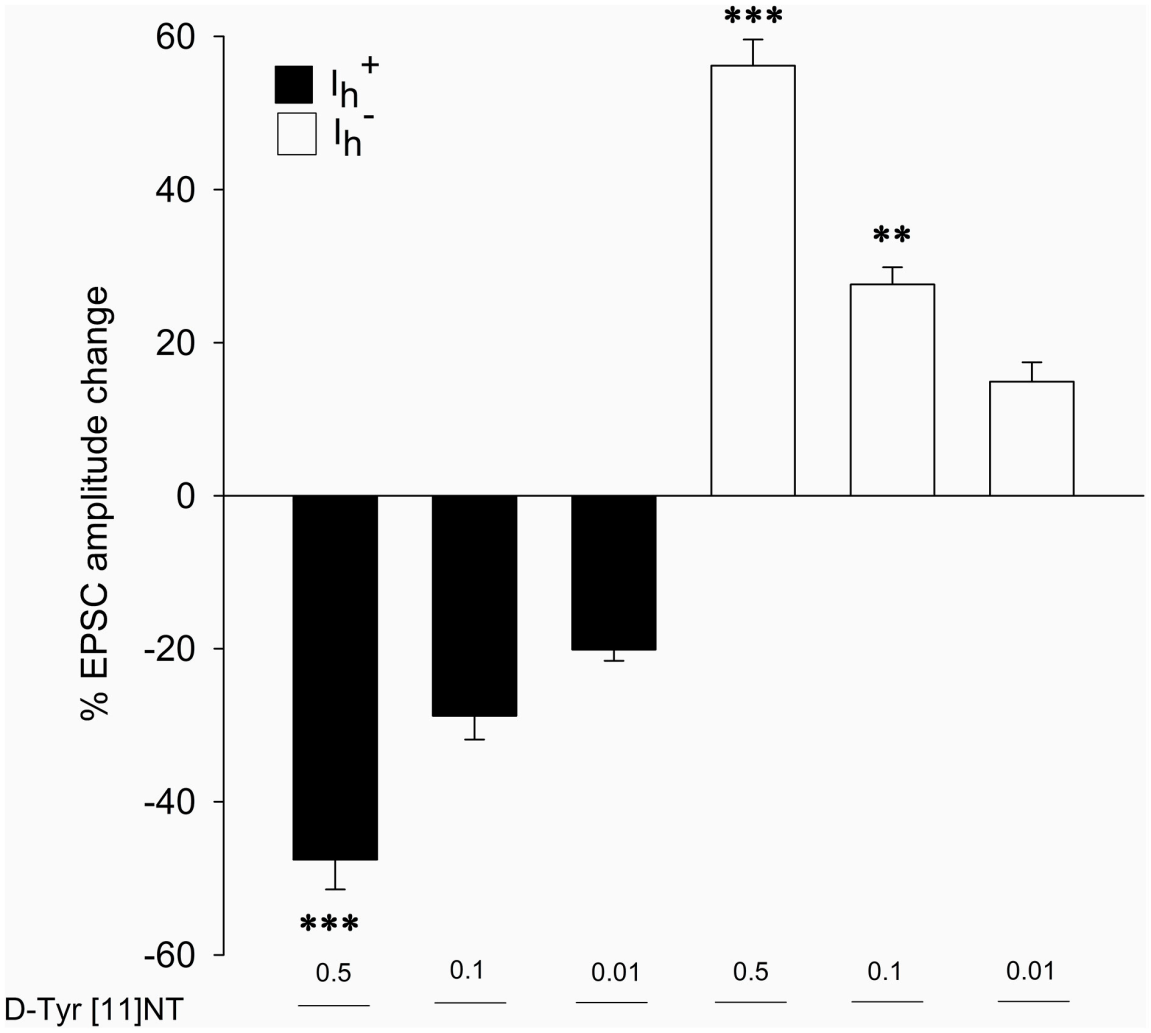
**Effects of [D-Tyr^**11**^]NT on glutamatergic EPSCs in ${\text{I}}_{\text{h}}^{+}$ and ${\text{I}}_{\text{h}}^{-}$ neurons**. Mean percent change in EPSC amplitude recorded in ${\text{I}}_{\text{h}}^{+}$ (black bar) and ${\text{I}}_{\text{h}}^{-}$ (white bar) following application of different concentrations of [D-Tyr^11^]NT. The number of neurons recorded at each concentration is as follow: 0.01 μM, *n* = 12 (${\text{I}}_{\text{h}}^{+}$ *n* = 6, ${\text{I}}_{\text{h}}^{-}$ *n* = 6); 0.1 μM, *n* = 12 (${\text{I}}_{\text{h}}^{+}$ *n* = 6, ${\text{I}}_{\text{h}}^{-}$ *n* = 6); 0.5 μM, *n* = 16 (${\text{I}}_{\text{h}}^{+}$ *n* = 7, ${\text{I}}_{\text{h}}^{-}$ *n* = 9). All concentrations of D-Tyr[11]NT are reported in μM. Asterisks indicate a statistically significant difference with the lowest concentration (^**^p < 0.05; ^***^p < 0.001). See text for details.

Representative traces of the evoked EPSCs from a single ${\text{I}}_{\text{h}}^{+}$ cell obtained before, during and after washout of 0.01 μM of [D-Tyr^11^]NT are shown in Figure 4. It can be seen that [D-Tyr^11^]NT attenuated the EPSC and that this effect was completely reversible. As shown in Figure 5 [D-Tyr^11^]NT produced a dose dependent reduction in the amplitude of the glutamatergic EPSCs in ${\text{I}}_{\text{h}}^{+}$ cells. At concentrations of 0.01, 0.1, and 0.5 μM, the mean decrease in EPSC amplitude was 20 ± 1.5% (*n* = 6), 29 ± 3% (*n* = 6), and 47 ± 4% (*n* = 7) respectively. A One-way ANOVA yielded significant effect [*F*_(2, 16)_ = 20.95, *p* < 0.001] and *post-hoc* test confirmed that the highest concentration produced a decrease that was significantly different than that produced by the lower concentration (*p* < 0.001).

**Figure 6:**
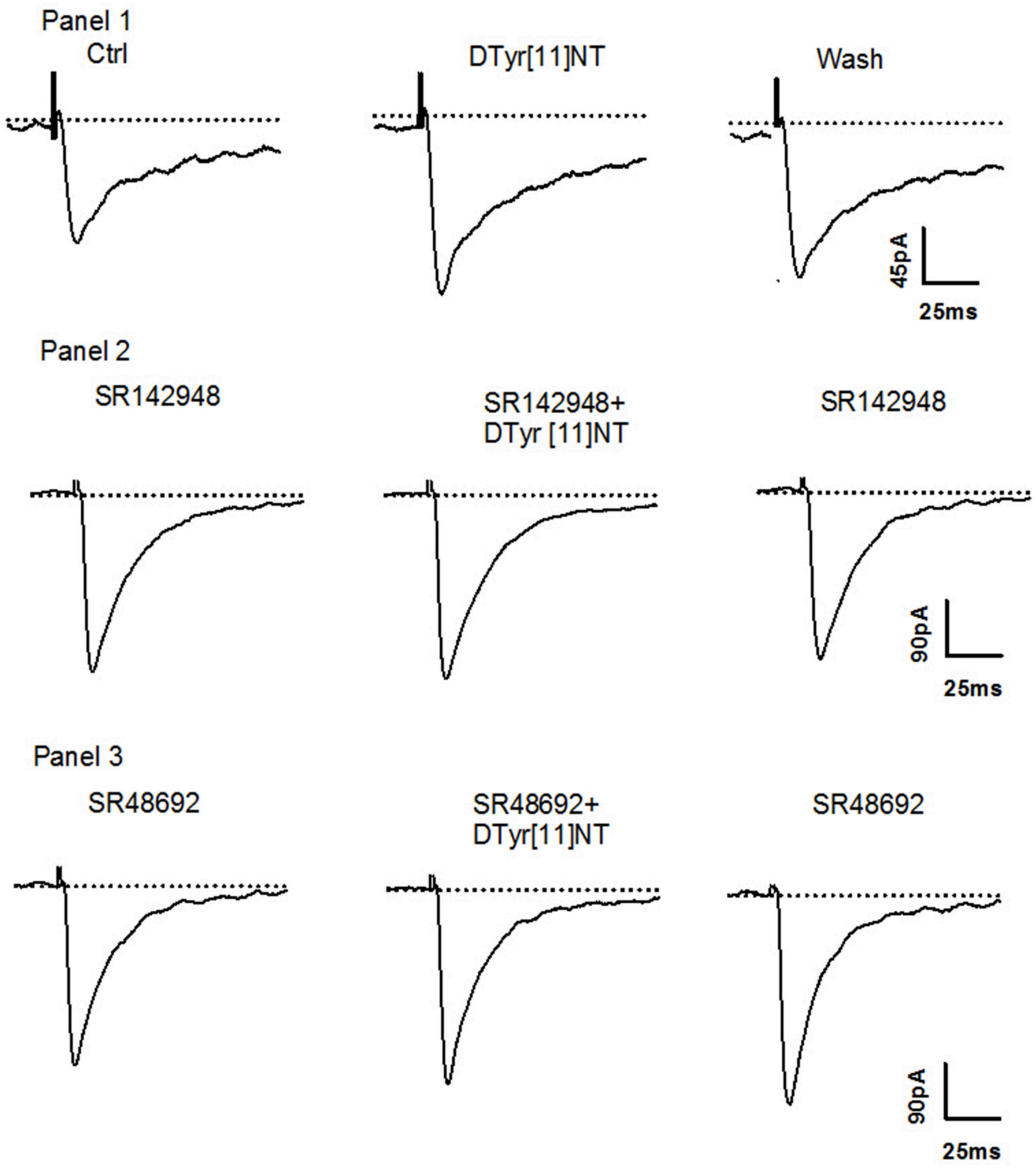
**Effect of [D-Tyr^**11**^]NT and antagonists on ${\text{I}}_{\text{h}}^{-}$ neurons**. **Panel 1:** Current traces of glutamatergic EPSC recorded during superfusion of [D-Tyr^11^]NT; control (1), with [D-Tyr^11^]NT (0.01 μM) (2) and following the washout of [D-Tyr^11^]NT (3) at a holding membrane potential of −70 mV in ${\text{I}}_{\text{h}}^{-}$ neurons (*n* = 6). **Panel 2:** Current traces of glutamatergic EPSC recorded during superfusion with SR142948 (0.1 μM) (1), with SR142948 and [D-Tyr^11^]NT (0.01 μM) (2) and with SR142948 following the washout of [D-Tyr^11^]NT (3) at a holding membrane potential of −70 mV in ${\text{I}}_{\text{h}}^{-}$ neurons (*n* = 5). **Panel 3:** Current traces of glutamatergic EPSC recorded during superfusion with SR48692 (0.1 μM) (1), with SR48692 and [D-Tyr^11^]NT (0.01 μM) (2) and with SR48692 following the washout of [D-Tyr^11^]NT (3) at a holding membrane potential of −70 mV in ${\text{I}}_{\text{h}}^{-}$ neurons (*n* = 5).

In ${\text{I}}_{\text{h}}^{-}$ cells, [D-Tyr^11^]NT produced a dose-dependent increase in the amplitude of the evoked EPSC. Representative traces obtained from a single ${\text{I}}_{\text{h}}^{-}$ cell illustrated in Figure 6 show that the enhancement effect of [D-Tyr^11^]NT was also reversible. The mean increase at concentrations of 0.01, 0.1, and 0.5 μM was 15 ± 2.5% (*n* = 6), 28 ± 2% (*n* = 6), and 56 ± 3.5% (*n* = 9) respectively (Figure 5). A One-way ANOVA yielded significant results [*F*_(2, 18)_ = 51.7, *p* < 0.001]; *post-hoc* test showed that there a significant different between the highest concentration and the two others (*p* < 0.001), and between the medium and the lowest concentration (*p* < 0.01).

### Effect of NTS antagonists in ${\text{I}}_{\text{h}}^{+}$ and ${\text{I}}_{\text{h}}^{-}$ neurons

**Figure 7:**
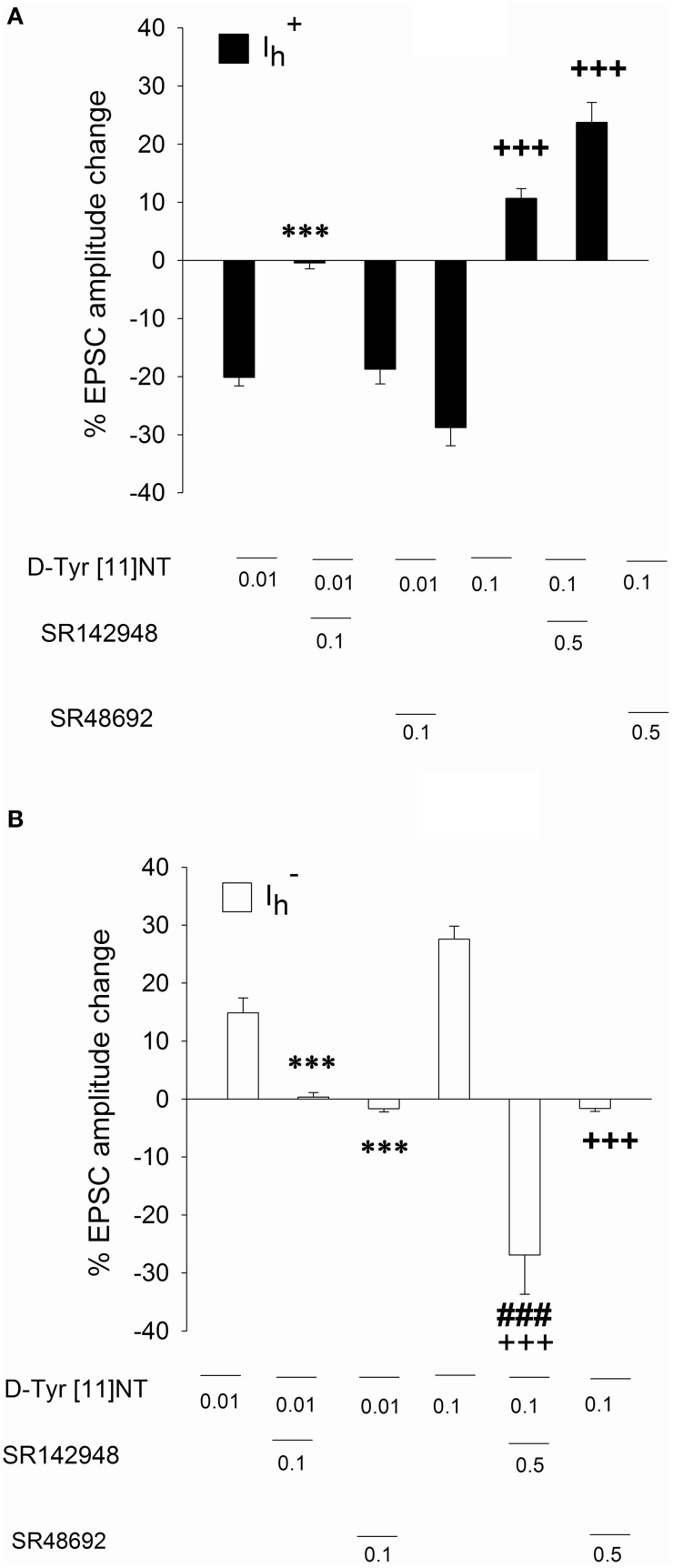
**Effect of SR142948 and SR48692 on glutamatergic EPSCs in ${\text{I}}_{\text{h}}^{+}$ and ${\text{I}}_{\text{h}}^{-}$ neurons**. Mean percent change in EPSC amplitude recorded in ${\text{I}}_{\text{h}}^{+}$ **(A)** and ${\text{I}}_{\text{h}}^{-}$ neurons **(B)** following application of [D-Tyr^11^]NT alone or in the presence of SR142948 or SR48692. The number of neurons recorded under each condition is as follow: ${\text{I}}_{\text{h}}^{+}$ neurons, [D-Tyr^11^]NT (0.01 μM, *n* = 6; 0.1 μM, *n* = 6); SR142948 (0.1 μM, *n* = 5; 0.5 μM, *n* = 4); SR48692 (0.1 μM, *n* = 4; 0.5 μM, *n* = 4); ${\text{I}}_{\text{h}}^{-}$ neurons, [D-Tyr^11^]NT (0.01 μM, *n* = 6; 0.1 μM, *n* = 6); SR142948 (0.1 μM, *n* = 5; 0.5 μM, *n* = 5); SR48692 (0.1 μM, *n* = 5; 0.5 μM, *n* = 4). All concentrations of D-Tyr[11]NT, SR142948, and SR48692 are reported in μM. Asterisks and crosses indicate a statistically significant difference with [D-Tyr^11^]NT alone at 0.01 μM and 0.1 μM respectively (^***^p < 0.001 with 0.01 μM; ^+++^*p* < 0.001 with 0.1 μM). The ^*###*^ sign indicates a statistical significant difference between SR142948 (0.5 μM) +D-Tyr[11]NT (0.1 μM) and SR48692 (0.5 μM) +D-Tyr[11]NT (0.1 μM). See text for details.

To identify the neurotensin receptor subtype(s) involved in the enhancement and attenuation effects of [D-Tyr^11^]NT on the evoked EPSCs in each cell population, we measured the EPSCs in the presence of SR142948 or SR48692. We found that in ${\text{I}}_{\text{h}}^{+}$ cells, SR48692 (0.5 μM) and SR142948 (0.5 μM) were both effective at blocking the decrease in EPSC amplitude produced by [D-Tyr^11^]NT (Figure 7, top panel). It can be noted that in the in the presence of this high concentration of the antagonists, [D-Tyr^11^]NT produced an enhancement of the EPSC amplitude. A One way ANOVA yielded a significant effect [*F*_(2, 11, 89.3)_ = *p* < 0.001] and *post-hoc* test confirmed that the EPSCs measured in the presence of the antagonist were significantly different than the EPSCs measured in the presence of [D-Tyr^11^]NT alone; there was also a significant difference between the effect of SR142948 and SR48692. Interestingly, different results were obtained when the concentration of the antagonist was reduced to 0.1 μM. At this concentration, SR142948 blocked the attenuation effect of [D-Tyr^11^]NT while SR48692 had no effect (Figures 4, 7). The ANOVA yielded significant effect [*F*_(2, 11)_= 37.7, *p* < 0.001] and *post-hoc* test confirmed that the mean EPSC measured in the presence of SR142948 was significantly different than that measured in the presence of [D-Tyr^11^]NT alone or SR48692 + [D-Tyr^11^]NT.

In ${\text{I}}_{\text{h}}^{-}$ cells, at the highest concentration (0.5 μM), SR48692 and SR142948 blocked the enhancement effect of [D-Tyr^11^]NT on the EPSC (Figure 7, bottom panel); in the presence of SR142948, [D-Tyr^11^]NT produced a large attenuation of the EPSC. The ANOVA yielded significant effect [*F*_(2, 14)_= 40.6, *p* < 0.001] and *post-hoc* test showed that the EPSC measured in the presence of SR142948 and SR48692 was significantly different than that measured in the presence of [D-Tyr^11^]NT alone. There was also a significant difference in EPSCs measured in the presence of SR142948 and SR48692, confirming that SR142948 led to a significant attenuation. Similarly to what we observed in ${\text{I}}_{\text{h}}^{+}$ cells, different results were obtained when the concentration of the antagonists was reduced to 0.1 μM. At this concentration, SR142948 and SR48692 similarly blocked the enhancement effect of [D-Tyr^11^]NT (Figures 6, 7). The ANOVA yielded significant effect [*F*_(2, 13)_= 18.5, *p* < 0.001] and *post-hoc* test confirmed that the mean EPSC measured in the presence of SR142948 and SR48692 were not different but were both different than that measured in the presence of [D-Tyr^11^]NT alone.

## Discussion

The main finding of this study is that activation of ventral midbrain NTS1 receptor induces a CPP and that this effect can be mediated, at least in part, through an enhancement of glutamatergic synaptic input in non-dopamine neurons in the VTA. Our results also show that [D-Tyr^11^]NT activates NTS2 receptors to reduce glutamatergic synaptic input to VTA dopamine and non-dopamine neurons. To our knowledge these findings constitute the first evidence that NT acts on two different NT receptor sub-types to modulate in an opposite manner glutamatergic neurotransmission in different populations of VTA neurons.

Previous studies have shown that ventral midbrain NT microinjection produces a rewarding effect as it sustains self-administration (Glimcher et al., 1987), enhances brain stimulation reward (Rompré et al., 1992) and induces a CPP (Glimcher et al., 1984). Consistently, we found that ventral midbrain microinjections of [D-Tyr^11^]NT dose-dependently induced a preference for the environment associated with the peptide. The induction of a CPP by [D-Tyr^11^]NT is consistent with many other results showing that this NT analog mimics several behavioral, physiological and neurochemical effects of NT (Jolicoeur et al., 1984; Donoso et al., 1986; al-Rodhan et al., 1991; Steinberg et al., 1995; Rompré, 1997). According to Glimcher et al. (1984), however, the induction of CPP by repeated ventral midbrain NT is not reproduced by an equimolar concentration of NT-(8-13); such a result was unexpected because NT-(8-13) displays a high affinity for the NTS1 (Kitabgi et al., 1980) and has been shown to be as effective as NT at inducing locomotor activity (Kalivas and Taylor, 1985; Steinberg et al., 1995), enhancing brain stimulation reward (Rompré and Gratton, 1993), increasing dopamine cell firing (Seutin et al., 1989; Shi and Bunney, 1991) and inducing polydipsia (Hawkins et al., 1989). One possible explanation is that Glimcher et al. (1984) compared the effectiveness of bilateral VTA microinjections of NT to equimolar unilateral microinjections of NT-(8-13); it could be that at the concentration used unilateral microinjections were insufficient to induce a CPP.

The induction of a CPP by [D-Tyr^11^] was blocked by SR48692 hence suggesting that it is mediated by NTS1 receptors. It has been shown that activation of NTS1 receptors expressed on dopamine neurons stimulates cell firing and dopamine release in brain regions known to play a key role in reward (Woulfe and Beaudet, 1989; Leonetti et al., 2004; St-Gelais et al., 2006; Thibault et al., 2011). Kempadoo et al. (2013) also showed that activation of VTA NTS1 receptors by local NT release reinforces operant responding. Altogether, this suggests that the induction of a CPP by [D-Tyr^11^]NT could be due to activation of NTS1 receptors expressed on VTA dopamine neurons. A role for NTS1 is in contradiction, however, with Glimcher et al's results showing a CPP is induced by repeated injections of NT-(1-11), a N-terminal fragment that fails to interact with the NTS1 receptor (Kitabgi et al., 1980). To our knowledge, no other studies have reported NT-like effects following central injection of NT-(1-11). That suggests that the conditioned rewarding effect of NT-(1-11) is NT-independent. This hypothesis is reinforced by an *in vitro* study reporting that NT-(1-11) dose-dependently inhibits cortisol production in cultured adrenocortical cells, an effect that is not reproduced by NT-(1-13) and not mediated by any of the known NT receptors (Sicard et al., 2006).

Conditioned place-preference is a learning process that involves neural plasticity. Drugs that induce a CPP, such as cocaine and morphine, induce lasting changes in VTA glutamatergic neurotransmission (Zweifel et al., 2008) and blockade of VTA glutamatergic receptors prevents cocaine- (Harris and Aston-Jones, 2003) and morphine-induced CPP (Harris et al., 2004). Kempadoo et al. (2013) have also shown that the rewarding effect of VTA NT release is associated with an enhancement of glutamatergic input to dopamine neurons. In order to determine whether the induction of a conditioned reward by [D-Tyr^11^]NT was related to a modulation of glutamatergic inputs to VTA neurons, we investigated the effect of [D-Tyr^11^]NT on glutamatergic EPSCs in different population of neurons distinguished with the presence or absence of an I_h_ current. Nearly, all dopaminergic neurons express an I_h_ current while ${\text{I}}_{\text{h}}^{+}$ neurons represent a subset of non-dopaminergic neurons; some GABA and glutamatergic neurons also express an I_h_ current (Lacey et al., 1989; Johnson and North, 1992; Margolis et al., 2006, 2012; Hnasko et al., 2012).

In the present study, we observed that bath application of varying concentrations of [D-Tyr^11^]NT generated a dose-dependent enhancement in the amplitude of glutamatergic EPSCs in ${\text{I}}_{\text{h}}^{-}$ neurons (non-dopamine neurons). This enhancement was most likely mediated by activation of NTS1 receptors as it was blocked by SR142948 and SR48692. These findings are in parallel with the behavioral results and suggest the action of NT on glutamatergic inputs to non-dopamine neurons may also play a key role in conditioned reward. Since the VTA contains a high density of NT terminals, it is thus possible that the effect of NT is not limited to glutamatergic inputs to dopamine neurons in this region (Jennes et al., 1982; Geisler and Zahm, 2006). Luo et al. (2010), for instance, showed that cocaine still induces a CPP in animals that had selective deletion of NMDA receptors onto dopamine neuron, and that this conditioned rewarding effect was NMDA-dependent.

In several limbic regions such as in the entorhinal cortex and the dentate gyrus of the hippocampus, activation of NTS1 receptors induces an excitatory effect and an increase in glutamate release (Rostène and Alexander, 1997; Antonelli et al., 2007, 2008); these effects are dependent on coupling to PLC, phosphokinase C (PKC) and Ca^2+^influx through L-type Ca^2+^ channels and activation of myosin light chain kinases respectively (Xiao et al., 2014; Zhang et al., 2015). Additionally, evidence of a facilitatory NTS1-NMDA receptor interaction at cortico-striatal glutamate terminals strengthens the role of NT in modulating glutamate release (Antonelli et al., 2004). Although, within the scope of our study, we were not able to identify the exact effector molecules mediating this action, association of NTS1 receptors to such downstream excitatory signaling cascades might have come into play.

When tested over the same range of concentrations, [D-Tyr^11^]NT generated a dose-dependent attenuation in the amplitude of glutamatergic EPSCs in ${\text{I}}_{\text{h}}^{+}$ neurons. This attenuation was most likely mediated by activation of NTS2 receptors as it was blocked by a low concentration of SR142948 but not SR48692. As mentioned previously, all VTA dopamine neurons are ${\text{I}}_{\text{h}}^{+}$ and that strongly suggests that [D-Tyr^11^]NT reduces glutamatergic inputs to at least a population of VTA dopamine neurons. In view of the evidence of a role for VTA dopamine in reward, and of the enhancement effect of NT on VTA dopamine impulse flow, these results were unexpected. They suggest that the action of [D-Tyr^11^]NT on glutamatergic inputs to VTA dopamine neurons through activation of NTS2 receptors is unlikely involved in the induction of a conditioned reward. In fact, the action of [D-Tyr^11^]NT on NTS2 should oppose its action on NTS1 and contribute to reduce its effectiveness at inducing a conditioned reward. This may explain why SR142948 which displays a similar affinity for NTS1 and NTS2 (Gully et al., 1997) was less effective than SR48692, a preferred NTS1 antagonist (Gully et al., 1995), at attenuating the induction of CPP.

There is also a large proportion of VTA GABA neurons that express an I_h_ current and it has been shown that GABA provides an inhibitory drive to dopamine neurons that is under the control of glutamate (Grace et al., 2007). An attenuation of glutamatergic input to these neurones is likely to enhance dopamine impulse flow resulting in reward and/or reward enhancement. If [D-Tyr^11^] NT is acting on NTS2 receptors to reduce glutamatergic EPSCs to these neurons, SR142948 would have been more effective than SR48692 at attenuating the induction of CPP; but as mentioned above, we observed the opposite.

The attenuation effect of [D-Tyr^11^]NT on glutamatergic EPSCs in ${\text{I}}_{\text{h}}^{+}$ neurons contrasts with the enhancement effect of NT and NT-(8-13) reported in previous studies (Kempadoo et al., 2013; Bose et al., 2015). Indeed both NT and NT-(8-13) enhance glutamatergic EPSC in ${\text{I}}_{\text{h}}^{+}$ neurons by activating NTS1 receptors. Kempadoo et al. (2013), however, observed a biphasic effect with NT-(8-13), an enhancement of NMDA EPSCs at a low concentration and an attenuation at a high concentration; the former but not the latter was blocked by SR48692 suggesting that the attenuation is not mediated by the NTS1 receptor. It thus appears that both NTS1 and NTS2 modulate glutamatergic inputs to ${\text{I}}_{\text{h}}^{+}$ neurons and that [D-Tyr^11^]NT has a predominant effect on the NTS2 receptor subtype. [D-Tyr^11^]NT has a higher affinity for NTS2 than NTS1 (Kitabgi et al., 1980; Labbé-Jullié et al., 1994) and activation of NTS2 receptors do not induce excitatory effects. For instance, activation of human NTS2 receptors expressed on CHO cell lines lacks the potential to elevate intracellular Ca^2+^ levels by mobilizing internal calcium reserves or accumulation IP3; it was rather associated with activation mitogen activated protein kinases (MAPK) that led to inhibition (Sarret et al., 2002). It could be that activation of NTS2 receptors on putative dopamine neurons enhances MAPK signaling and produces a reduction in glutamatergic signaling. Although there is no evidence reported in the literature that [D-Tyr^11^]NT can generate a NTS1- or NTS2-mediated biased signalization, it has been recently reported that modified analogs of NT-(8-13) at the 11 amino acid position displayed NTS1-mediated biased activation of G_α*q*_ and β-arrestin signaling pathways (Fanelli et al., 2015).

The 11th position substitution in [D-Tyr^11^]NT by a D-tyrosine residue makes it more resistant to cleavage by endopeptidases (Checler et al., 1983). In fact, after an intracerebroventricular injection of NT, 98% of the NT was cleared and degraded in brain tissues during a 30 min period after the injection. Under the same conditions, 33% of [D-Tyr^11^]NT was retained, suggesting a half-life 1.5 times greater than that of NT (Checler et al., 1983). Owing to the relatively stable metabolic profile of [D-Tyr^11^]NT it is possible that the reduction in EPSC observed by Kempadoo et al. (2013) with higher concentrations of NT8-13 reflects that produced by the lower concentrations of [D-Tyr^11^]NT used in the present study.

Interestingly, in the presence of a high concentration (0.5 μM) of SR142948, but not SR48692, [D-Tyr^11^]NT produced an opposite, significant inhibition, of glutamatergic EPSCs in ${\text{I}}_{\text{h}}^{-}$ neurons. This could possibly arise because at this concentration, SR142948 interacts with an NT receptor subtype other than NTS1 and NTS2 (possibly NTS3). The NTS3 protein and its mRNA are present in VTA and are mainly expressed on cell bodies and dendrites. According to Mazella et al. (1998), the NTS3 receptor is nearly insensitive to SR48692. Others, however, reported that SR142948 is effective at blocking the NTS3-mediated growth response to NT in cancer cells (Dal Farra et al., 2001); that would rather exclude a role for this receptor in the opposite effect of [D-Tyr^11^]NT on EPSC in the presence of SR142948. In ${\text{I}}_{\text{h}}^{+}$ neurons, the presence of a high concentration of SR142948 and SR48692 had the same impact; [D-Tyr^11^]NT enhanced the EPSC, an effect opposite to what was observed when it was infused alone. As mentioned previously, it remains unclear why the antagonists produce such a reverse effect.

## Author contributions

PR and KR designed the behavioral experiments; KR carried out the behavioral experiments and analyzed the data with PR. RW and PB designed the electrophysiological experiments; PB carried out the electrophysiological experiments and analyzed the data with RW and PR. All authors contributed and approved the final version of the manuscript.

## Funding

Funding for this study was provided by Canadian Institutes of Health Research (Grant #102572 to PR and RW), Natural Sciences and Engineering Research Council of Canada (Grant # 184095-2009 to RW) and Fonds de Recherche Santé Québec.

### Conflict of interest statement

The authors declare that the research was conducted in the absence of any commercial or financial relationships that could be construed as a potential conflict of interest.
